# Prognostic value of a modified lymph node ratio for combined colorectal cancer tumour deposits: a population-based study

**DOI:** 10.3389/fonc.2026.1910628

**Published:** 2026-09-03

**Authors:** Mingming Song, Feihong Wu, Yixin Heng, Tong Nie, Zhihao Liu, Jiaxin Xu, Xiaoyu Wu, Xiaoling Zhi, Wanrui Zhang, Zhixiang Yang, Menghui Dai, Jiaqi Wang, Gang Hu

**Affiliations:** 1 Department of General Surgery, The Second People’s Hospital of Hefei Affiliated to Bengbu Medical University, Hefei, China; 2 Department of General Surgery, The Second People’s Hospital of Hefei, Hefei, China; 3 Department of Urology, Harbin Medical University Cancer Hospital, Harbin, China; 4 Department of Radiology, Union Hospital, Tongji Medical College, Huazhong University of Science and Technology, Wuhan, China; 5 Hubei Provincial Clinical Research Center for Precision Radiology & Interventional Medicine, Wuhan, China; 6 Hubei Province Key Laboratory of Molecular Imaging, Wuhan, China; 7 Department of General Surgery, The First Affiliated Hospital of Shihezi University, Shihezi, China; 8 China Medical University, Shenyang, China; 9 Department of Gastrointestinal Surgery, Union Hospital, Tongji Medical College, Huazhong University of Science and Technology, Wuhan, China; 10 Cancer center, Union Hospital, Tongji Medical College, Huazhong University of Science and Technology, Wuhan, China

**Keywords:** colorectal cancer, lymph node ratio, nomogram, prognosis, tumor deposits

## Abstract

**Background:**

Tumour deposits (TDs) without metastatic lymph nodes are classified as N1c in the 8th edition of the AJCC staging manual. However, the prognostic value of TDs and lymph node ratio (LNR) in stage III colorectal cancer (CRC) remains unclear. This study aimed to compare the prognostic significance of TD count and LNR with the newly developed modified lymph node ratio (mLNR), and to create a prognostic nomogram integrating mLNR for stage III CRC patients.

**Methods:**

Using the SEER database, a training cohort of 25,689 stage III CRC patients was identified, alongside an external validation cohort of 563 patients from a hospital in China. Modified lymph node ratio (mLNR) was defined by combining TD count and LNR. The effects of TDs, LNR, and mLNR on cancer-specific survival (CSS) and overall survival (OS) were evaluated using Kaplan-Meier curves and Cox regression models. A nomogram incorporating mLNR was developed for prognosis prediction.

**Results:**

In both cohorts, TDs positivity was associated with poorer CSS (all P < 0.001). Multiple TDs correlated with worse CSS than a single TD (training: HR, 1.978; validation: HR, 1.805). High mLNR indicated a significantly higher risk of CRC-specific death (training: HR, 2.421; validation: HR, 4.60). The nomogram incorporating mLNR demonstrated good discrimination and calibration in both cohorts.

**Conclusions:**

mLNR is an independent prognostic factor for stage III CRC. The nomogram combining mLNR can effectively identify high-risk patients, aiding treatment decisions.

## Introduction

Colorectal cancer (CRC) is the third most prevalent cancer worldwide and has the second highest mortality rate of all cancers (1–3). In clinical practice, the assessment of prognosis in patients with CRC is based on the American Joint Committee on Cancer (AJCC) TNM staging system, which is of paramount importance in determining treatment decisions and survival prognosis for patients. Nevertheless, the system’s capacity to predict the prognosis of CRC remains limited, particularly in stage III patients. This is because stage III CRC encompasses heterogeneous combinations of tumour invasion depth and regional lymph node involvement. For example, T3N1 and T2N2b tumours can both be classified as stage IIIB, although the former is characterised by deeper local invasion with a relatively lower nodal burden, whereas the latter has shallower invasion but more extensive lymph node metastasis. These different patterns of tumour spread may be associated with different clinical outcomes, resulting in residual prognostic heterogeneity even within the same TNM stage subgroup (4). Consequently, there is a necessity to explore new prognostic markers with a view to further refining staging and optimising treatment strategies.

In recent years, tumour deposits (TDs) have gradually attracted extensive attention from researchers as an important factor in the prognostic assessment of colorectal cancer. According to the WHO classification, TDs are discrete macroscopic or microscopic nodules of carcinoma in the pericolorectal adipose tissue within the lymphatic drainage area of the primary tumour; they are discontinuous from the primary tumour and show no histological evidence of residual lymph-node tissue or identifiable vascular or neural structures (5, 6). The presence of TDs suggests that tumour cells may have spread to lymphatic drainage areas, and despite not meeting the criteria for LNM, its potential metastatic and biologically malignant characteristics still significantly affect patient prognosis. Whilst the current TNM staging system classifies TDs without LNM as N1c, this classification does not fully reflect the independent significance of TDs in colorectal cancer prognosis (7, 8). The presence of TDs is either staged as N1c in the absence of LNM, or is entirely ignored in the presence of LNM, with the result that its prognostic significance is completely ignored (9). Even in N1c, the presence or absence of TDs was recorded alone, while the number of TDs was not taken into account. This can also lead to an underestimation of the prognostic value of TDs. When the TDs status of patients with N1a/b and N2a/b is ignored, and the number of TDs in patients with N1c, the risk for patients with stage III may be underestimated, which could result in inadequate adjuvant therapy (10). Recent studies have demonstrated a substantial correlation between the status and number of TDs and patient survival (11). Furthermore, it has been posited that CRC patients with a greater number of TDs are likely to demonstrate a higher incidence of local recurrence and an inferior overall survival rate (8, 12).

In addition to the TDs, the lymph node ratio (LNR) is considered a significant factor in the prognostic assessment of patients diagnosed with colorectal cancer. LNR is defined as the ratio of the number of LNM to the total number of lymph nodes resected. It has been demonstrated that LNR is a significant marker of tumour load and is strongly associated with survival prognosis in patients diagnosed with colorectal cancer (13–15). In contrast to relying exclusively on the number of LNM, LNR has the capacity to offer a more comprehensive reflection of the density of LNM and the extent of tumour spread, thereby enhancing the accuracy of prognostic prediction. A number of studies have demonstrated that elevated LNR is associated with diminished cancer-specific survival (CSS) and overall survival (OS). Furthermore, LNR has been determined to possess independent prognostic significance in patients diagnosed with stage III CRC (16–18).

In the present study, we evaluated the prognostic value of TD status, TD count, and LNR in a training cohort derived from the Surveillance, Epidemiology, and End Results (SEER) database and an external validation cohort from a local hospital. We then developed a modified lymph node ratio (mLNR) by integrating the TD-count and LNR categories and constructed an mLNR-based nomogram to predict CSS and OS in patients with stage III CRC. The model was externally validated in the local hospital cohort.

## Materials and methods

### Patients and data sources

The present study comprised two cohorts: a training cohort and a validation cohort. We extracted the data of patients with CRC from the SEER database from 2010 to 2020 by applying SEER * stat (version 8.4.4). The validation cohort comprised eligible patients from Wuhan Union Medical College Hospital between 2014 and 2018. The inclusion criteria were as follows:(1) patients with stage III CRC; (2) patients who underwent surgical resection at the primary site, primary site ICD-O-3 codes: C18.0, C18.1, C18.2, C18.3, C18.4, C18.5, C18.6, C18.7, C18.8, C18.9, C19.9, and C20.9; (3) for the SEER cohort, patients with pathologically confirmed malignant CRC diagnosed between 2010 and 2020, identified using ICD-O-3 morphology codes (behaviour code/3), including adenocarcinoma, NOS (8140); other carcinoma or adenocarcinoma histological subtypes (8010, 8020, 8141–8144, 8210, 8211, 8255, 8260–8263, 8310, 8440, 8460, 8550, and 8560); mucinous adenocarcinoma and related mucinous subtypes (8470–8472, 8480, and 8481); and signet-ring cell carcinoma (8490). The exclusion criteria comprised the following: (1) patients younger than 18 years of age; (2) patients with survival time of less than 1 month; (3) patients who received neoadjuvant therapy, because treatment-related downstaging could affect postoperative pathological T and N stages, TD detection, and lymph-node assessment; (4) patients with omission of critical information on lymph nodes and TDs; (5) individuals with incomplete or incorrect clinicopathological information.

The present study was conducted in accordance with the SEER data use agreement, and patient informed consent was not required given the anonymised, de-identified data in the SEER database. The study was approved by the Ethics Committee of Wuhan Union Medical College Hospital (No. 2018-S377). The ethics committee waived informed consent due to the retrospective nature of the study.

### Variables and outcomes

The clinicopathological factors investigated in this study included date of diagnosis, gender, age, tumour laterality, tumor size, tumor number, grade, T stage, N stage, carcinoembryonic antigen (CEA), perineural invasion (PNI), TDs, number of metastatic lymph nodes, total number of lymph nodes examined (examined N), and chemotherapy status. In the external validation set, Chinese patients were considered positive for CEA levels greater than 5 ng/mL (19). Right-sided tumours included tumours of the cecum, ascending colon, transverse colon and hepatic flexure of the colon; left-sided tumours were defined as tumours of the rectum, rectosigmoid junction, sigmoid colon, descending colon and splenic flexure of the colon (20). OS is measured from the date of diagnosis to the date of death from any cause, while CSS is defined as the time from diagnosis to death from cancer (21). The survival outcomes in the training set were derived from the SEER database, while the survival outcomes in the validation set were derived from telephone-based active follow-up and patient post-operative review.

### Creation of modified LNR

In order to enhance the precision of LNR in prognostic assessment and to more accurately reflect the prognostic value of TDs counts in CRC patients, a novel mLNR was developed, incorporating TDs counts and LNR. Firstly, patients were divided into three categories according the number of TDs: no TD, one TD and multiple TDs. Subsequently, by combining the TD-count and LNR categories, mLNR were created, which were then simplified into three risk categories: low, intermediate, and high risk. Patients with no TD and low LNR (≤0.37) were assigned to the low-risk group, whereas those with multiple TDs and high LNR (>0.37) were assigned to the high-risk group. All remaining combinations—no TD and high LNR, one TD with either LNR category, and multiple TDs with low LNR—were classified as intermediate risk. The detailed process of creating the mLNR is outlined in **Table 1**.

**Table 1:** Detailed demonstration of mLNR creation.

Number of TD	LNR	mLNR
0	Low (≤ 0.37)	Low
	High (> 0.37)	Intermediate
1	Low (≤ 0.37)	Intermediate
	High (> 0.37)	Intermediate
>1	Low (≤ 0.37)	Intermediate
	High (> 0.37)	High

LNR was categorised as low (≤0.37) or high (>0.37). mLNR was classified into low-, intermediate-, and high-risk groups according to the combinations shown. TD, tumour deposit; LNR, lymph node ratio; mLNR, modified lymph node ratio.

### Statistical analysis

All statistical analyses were conducted utilising SPSS 26.0 software and R-software version 4.3.0. A comparison was made of the demographic and tumour characteristics of the training and validation cohorts, respectively, and of the two groups according to TDs status, using the χ2 test. The optimal LNR cut-off value of 0.37 was determined using X-tile (version 3.6.1; Yale University School of Medicine, New Haven, CT, USA). Patients were categorised into a low-LNR group (LNR ≤ 0.37) and a high-LNR group (LNR>0.37). The strength of association of clinicopathological factors with CSS and OS was clarified using the likelihood ratio test. Survival analysis was performed using the Kaplan-Meier method and log-rank test.

Variables significantly associated with survival were screened by univariate and multivariate Cox regression analyses. Those with P < 0.01 were then entered into the Cox proportional risk model to construct a predictive model for CRC patients. This was presented in the form of a nomogram. The predicted outcomes of interest were 1-, 3-, and 5-year total OS and CSS. In order to assess the predictive power of the model, time-dependent receiver operating characteristic (ROC) curves and concordance index (C-index) were implemented. The area under the ROC curve (AUC) and the C-index ranged from 0.5 to 1, with a value above 0.7 indicating good predictive power. Calibration curve plots were used to assess the degree of difference between predicted and actual risk. Decision curve analysis (DCA) was also performed to assess the clinical benefit and usefulness of the constructed predictive models. It is imperative to note that all statistical tests employed in this study were two-sided, and a P value < 0.05 was considered statistically significant.

## Results

### Patient clinicopathologic characteristics

**Figure 1:**
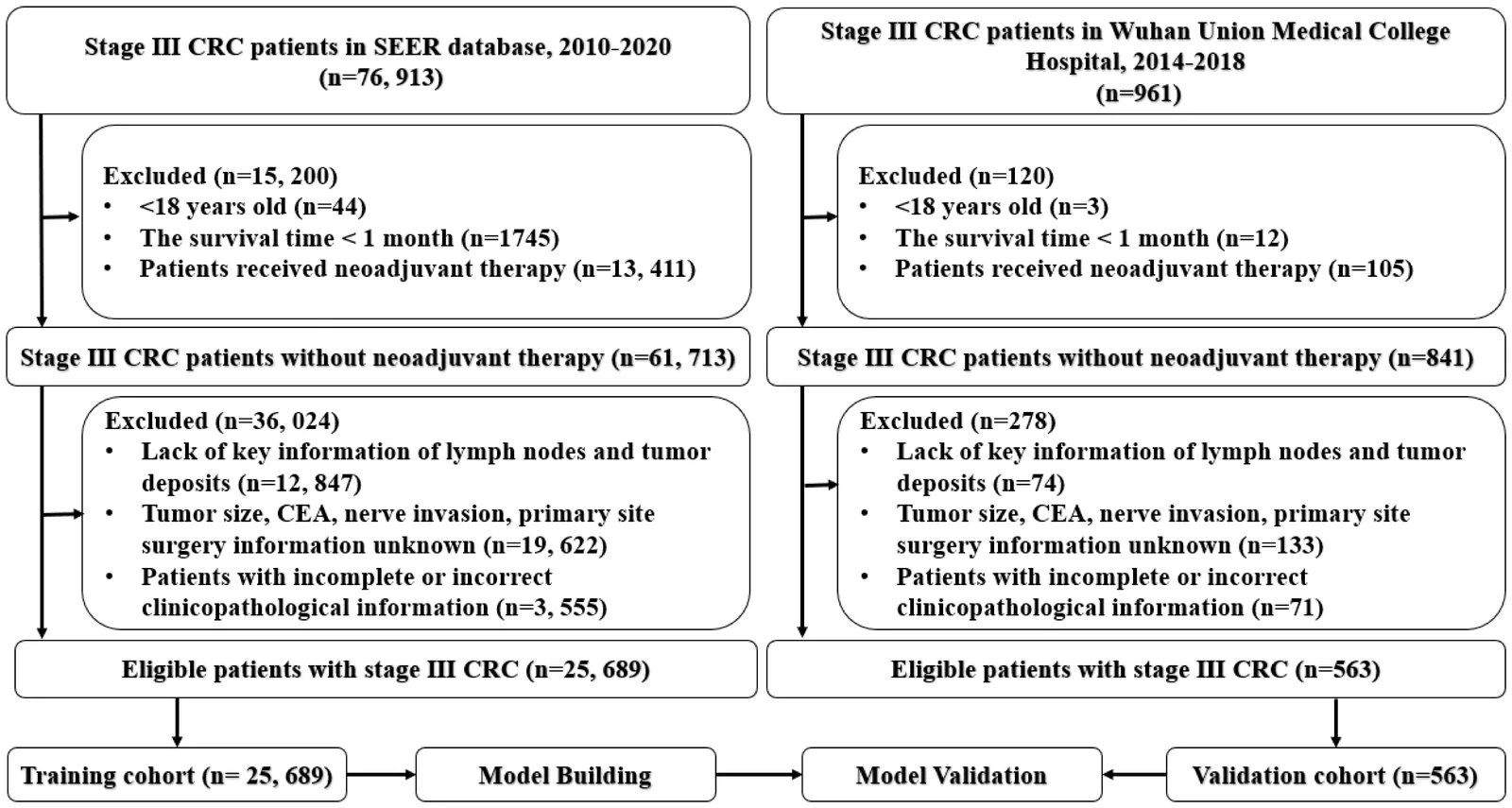
Flowchart of the population.

A total of 25,689 eligible CRC patients were collected from the SEER database as a training cohort, including 5,136 patients with TDs and 20,553 patients without TDs (**Figure 1**). The external validation cohort comprised 563 CRC patients, including 144 patients experiencing TDs and 419 patients not experiencing TDs, respectively (**Figure 1**). The median age of the training cohort was 65 years (range, 18–96 years), and the median age of the validation cohort was 59 years (range, 21–97 years). The 3-year and 5-year CSS for the training cohort were found to be 82.8% and 74.8%, correspondingly, whereas for the validation cohort, these rates were 79.2% and 67.6%. The 3- and 5-year OS were 76.6% and 65.3% for the training cohort, and 76.3% and 64.1% for the validation cohort, respectively. The baseline characteristics of patients classified according to TDs are shown in **Table 2**. In the training cohort, no statistically significant differences were observed between the TDs (+) and TDs (-) groups with regard to gender, age, and chemotherapy (all P > 0.05). In both the training cohort and the validation cohort, stage III CRC patients with TDs exhibited a higher frequency of T3–4 status, were CEA-positive, had PNI, had higher LNR, and had tumors localized more commonly in the left‐sided colon compared to patients without TDs (all P < 0.05). Additionally, patients with TDs in the training cohort exhibited larger tumours, poorer differentiation, a higher proportion of N2 stages, and a lower number of examined N (all P < 0.05).

**Table 2:** Patient’s baseline characteristics by tumor deposits.

Characteristic	Training cohort			Validation cohort		
	TD-negative N = 20553(%)	TD-positive N = 5136(%)	P-value	TD-negative N = 419(%)	TD-positive N = 144(%)	P-value
Sex			0.304			0.484
Male	10692 (52.0)	2713 (52.8)		248 (59.2)	90 (62.5)	
Female	9861 (48.0)	2423 (47.2)		171 (40.8)	54 (37.5)	
Age (years)			0.180			0.996
< 65	10522 (51.2)	2683 (52.2)		296 (70.6)	102 (70.8)	
≥ 65	10031 (48.8)	2453 (47.8)		123 (29.4)	42 (29.2)	
Laterality			<0.001			0.194
Left	12660 (61.6)	3480 (67.8)		309 (73.7)	114 (79.2)	
Right	7893 (38.4)	1656 (32.2)		110 (26.3)	30 (20.8)	
Tumor number			0.001			0.607
1	14980 (72.9)	3863 (75.2)		378 (90.2)	132 (91.7)	
> 1	5573 (27.1)	1273 (24.8)		41 (9.8)	12 (8.3)	
Size			<0.001			0.189
< 5 cm	13442 (65.4)	3218 (62.7)		315 (75.2)	116 (80.6)	
≥ 5 cm	7111 (34.6)	1918 (37.3)		104 (24.8)	28 (19.4)	
Grade			0.005			0.540
Well/moderate	16307 (79.3)	3983 (77.6)		358 (85.4)	126 (87.5)	
Poor/undifferentiated	4246 (20.7)	1153 (22.4)		61 (14.6)	18 (12.5)	
T stage			<0.001			0.047
T1-T2	3360 (16.3)	352 (6.9)		51 (12.2)	9 (6.3)	
T3-T4	17193 (83.7)	4784 (93.1)		368 (87.8)	135 (93.8)	
N stage			<0.001			<0.001
N1	14609 (71.1)	3404 (66.3)		288 (68.7)	74 (51.4)	
N2	5944 (28.9)	1732 (33.7)		131 (31.3)	70 (48.6)	
Examined N			<0.001			0.507
<12	2743 (13.3)	810 (15.8)		63 (15.0)	25 (17.4)	
≥12	17810 (86.7)	4326 (84.2)		356 (85.0)	119 (82.6)	
Chemotherapy			0.561			0.730
No	5695 (27.7)	1444 (28.1)		188 (44.9)	67 (46.5)	
Yes	14858 (72.3)	3692 (71.9)		231 (55.1)	77 (53.5)	
CEA			<0.001			0.001
Negative	12320 (59.9)	2766 (53.9)		229 (54.7)	55 (38.2)	
Positive	8233 (40.1)	2370 (46.1)		190 (45.3)	89 (61.8)	
PNI			<0.001			<0.001
Absent	17419 (84.8)	3613 (70.3)		303 (72.3)	74 (51.4)	
Present	3134 (15.2)	1523 (29.7)		116 (27.7)	70 (48.6)	
LNR			<0.001			0.002
Low	18270 (88.9)	4370 (85.1)		359 (85.7)	107 (74.3)	
High	2283 (11.1)	766 (14.9)		60 (14.3)	37 (25.7)	

TD, tumor deposits; Examined N, total examined lymph nodes; CEA, carcino-embryonic antigen, PNI, perineural invasion; LNR, lymph node ratio.

### Prognostic value of TDs, LNR, and mLNR

**Figure 2:**
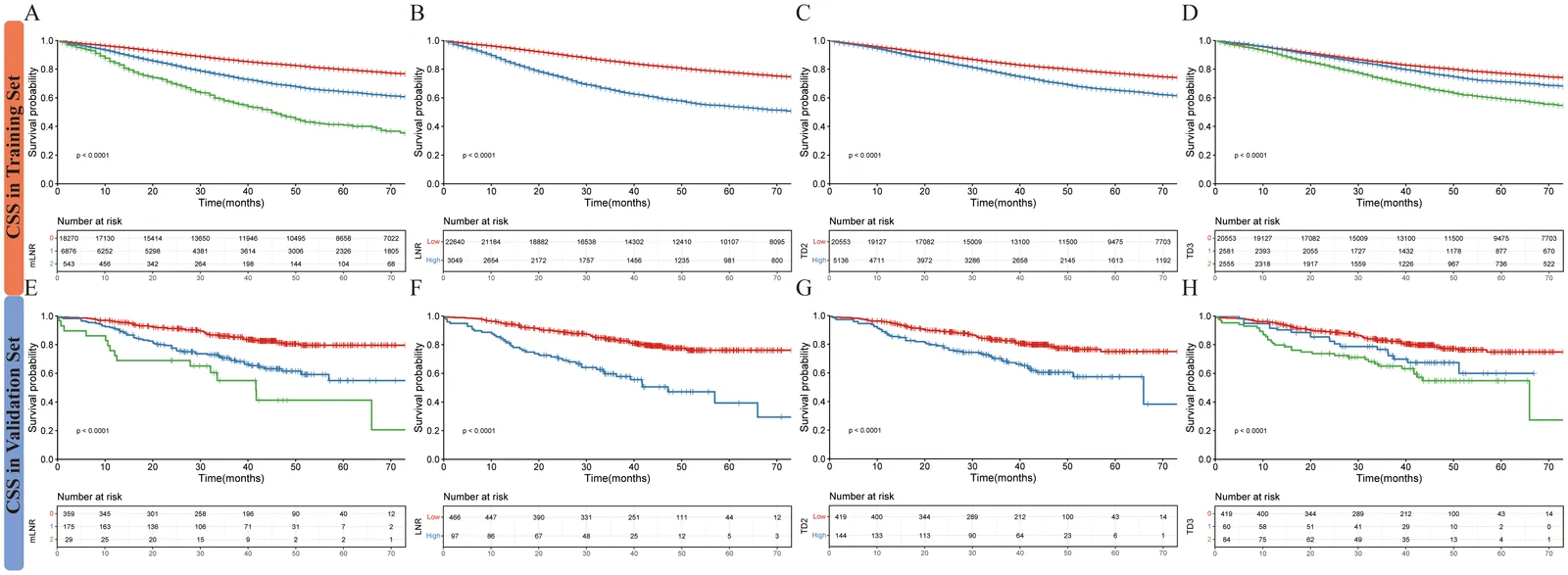
Kaplan-Meier curves for cancer-specific survival (CSS) stratified by different biomarkers in the training set **(A–D)** and validation set **(E–H)**. **(A, E)** Kaplan-Meier curves stratified by modified lymph node ratio (mLNR); **(B, F)** Kaplan-Meier curves stratified by lymph node ratio (LNR); **(C, G)** Kaplan-Meier curves stratified by the presence or absence of tumor deposits (TD; binary classification, TD2); **(D, H)** Kaplan-Meier curves stratified by the number of tumor deposits (TD: 0, 1, or >1; ternary classification, TD3). TD represents tumor deposits.

TD positivity and high LNR were associated with poorer CSS and OS in both cohorts (**Figure 2**; **Supplementary Figure 1**; all P < 0.001). We next evaluated TD count by classifying patients as having no TDs, a single TD, or multiple TDs. Increasing TD count was associated with poorer CSS (**Figures 2D, H**; all P < 0.001) and OS (**Supplementary Figures 1D, H**; all P < 0.001) in both cohorts. Compared with patients without TDs, those with multiple TDs had 4.09 times the hazard of CRC-specific death in the training cohort (HR, 4.09; 95% CI, 3.54–4.72; P < 0.001) and 4.60 times the hazard in the validation cohort (HR, 4.60; 95% CI, 2.88–7.35; P < 0.001). Multiple TDs also conferred poorer CSS and OS than a single TD (CSS: training cohort, HR, 1.978; 95% CI, 1.745–2.242; P < 0.001; validation cohort, HR, 1.805; 95% CI, 1.020–3.194; P = 0.043; OS: training cohort, HR, 1.806; 95% CI, 1.614–2.021; P < 0.001; validation cohort, HR, 1.861; 95% CI, 1.087–3.184; P = 0.023) (**Supplementary Figure 2**).

We then assessed mLNR, which integrates the TD-count and LNR categories. mLNR classified patients into low-, intermediate-, and high-risk groups with progressively poorer CSS. The corresponding 5-year CSS rates were 79.6%, 64.1%, and 40.9% in the training cohort (**Figure 2A**) and 76.4%, 55.0%, and 30.4% in the validation cohort (**Figure 2E**). OS showed a similar pattern in both cohorts (**Supplementary Figures 1A, E**).

**Figure 3:**
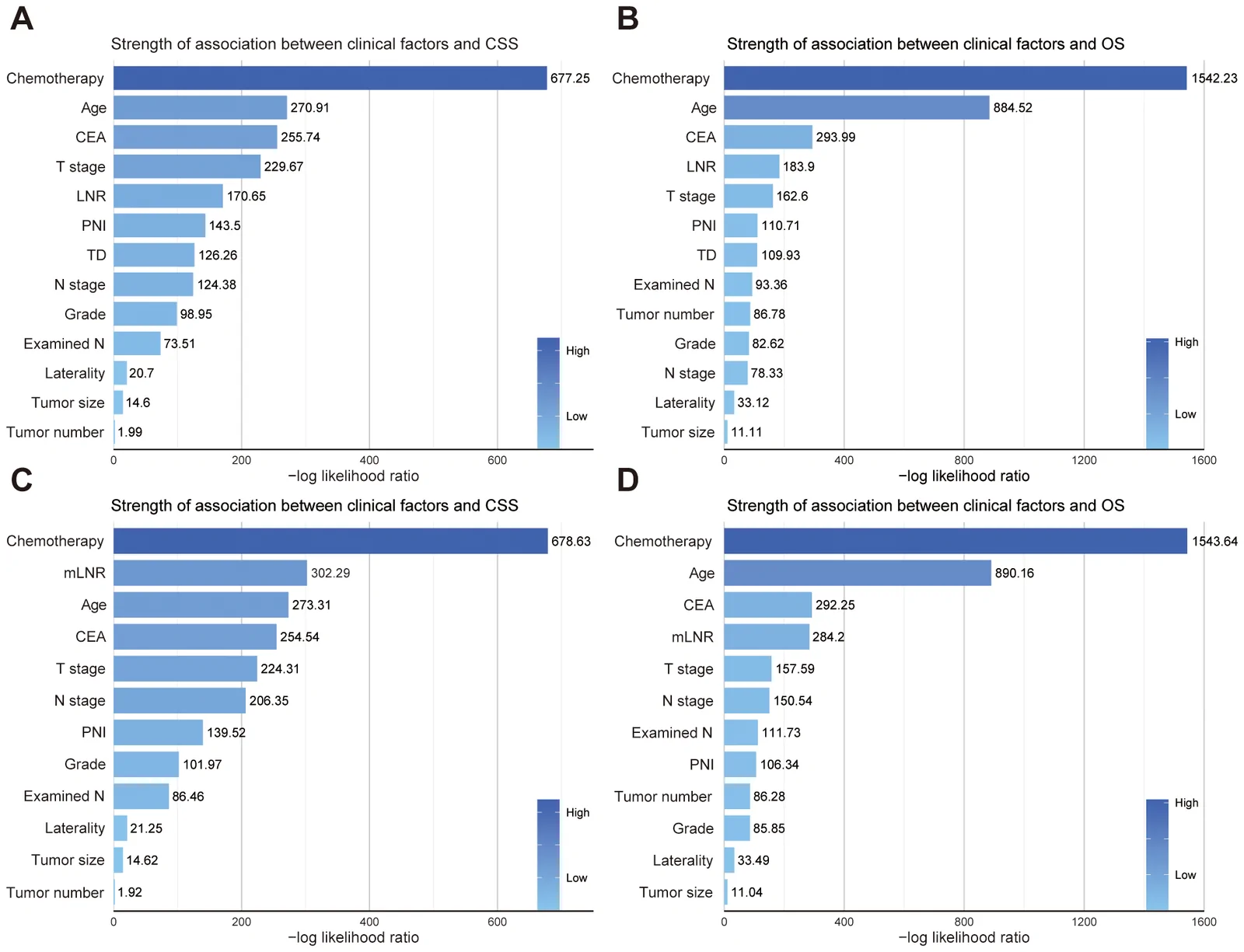
Strength of association between clinical variables and survival outcomes in the training set. Panels **(A, C)** depict the association strength with cancer-specific survival (CSS), while Panels **(B, D)** illustrate the relationship with overall survival (OS). Panels **(A, B)** are based on traditional prognostic variables, whereas Panels **(C, D)** incorporate the modified lymph node ratio (mLNR), an integrative metric combining tumor deposits (TD) and lymph node ratio (LNR). The comparative analysis highlights the prognostic significance of mLNR, suggesting its potential to refine survival stratification and enhance predictive accuracy. CSS, cancer-specific survival; OS, overall survival; TD, tumor deposits; LNR, lymph node ratio; mLNR, modified lymph node ratio; PNI, perineural invasion; CEA, carcinoembryonic antigen.

Likelihood ratio analyses further showed that mLNR had a stronger association with survival than either LNR or TDs alone. For CSS, LNR and TDs ranked fifth and seventh among the evaluated variables, with log likelihood ratios of 170.65 and 126.26, respectively (**Figure 3A**), whereas mLNR ranked second, after chemotherapy, with a log likelihood ratio of 302.29 (**Figure 3C**). A similar pattern was observed for OS: mLNR showed a stronger association with OS than either LNR or TDs alone (log likelihood ratio: mLNR, 284.20; LNR, 183.90; TDs, 109.93; **Figures 3B, D**).

### mLNR-based nomogram construction

**Figure 4:**
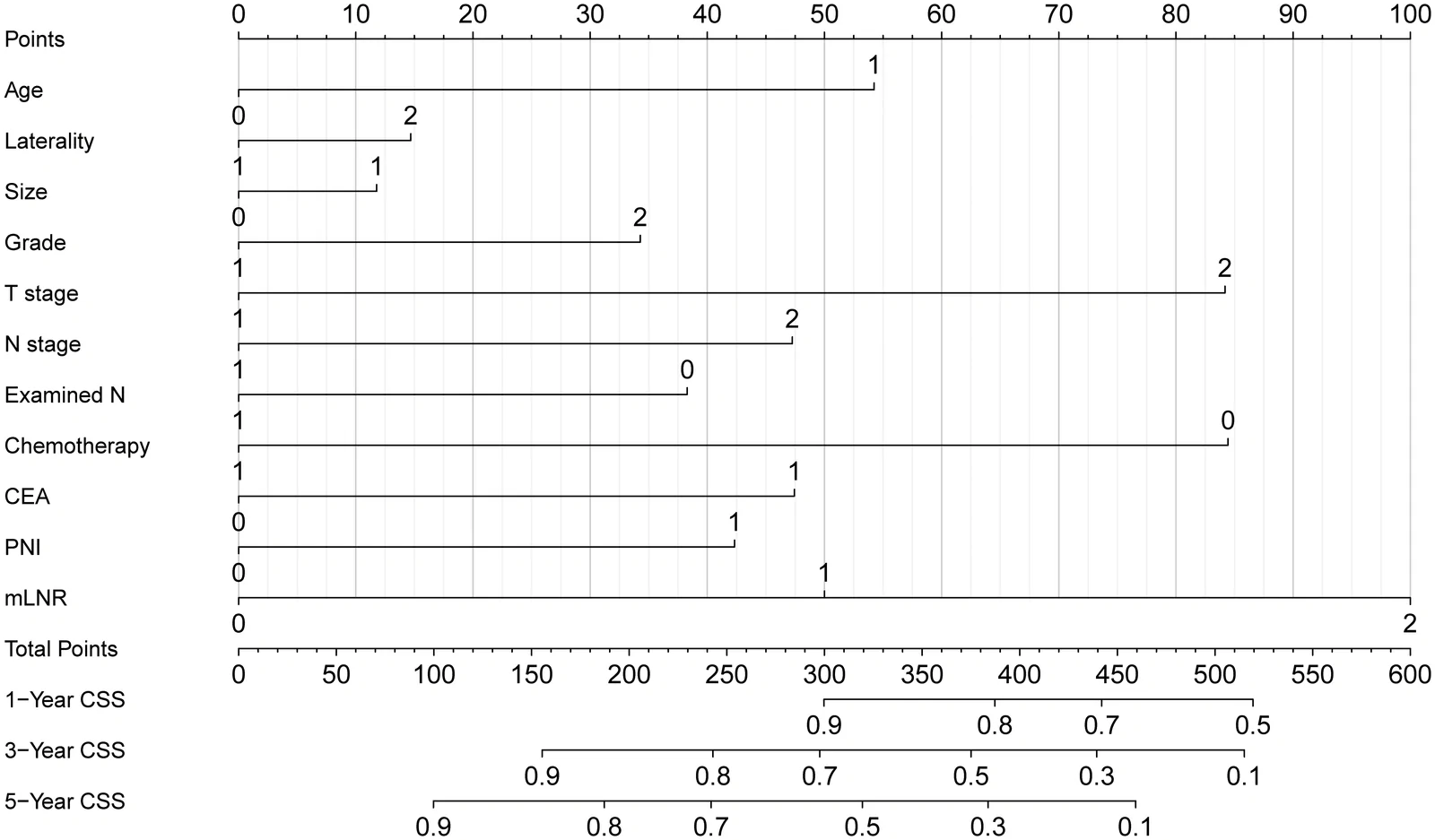
Nomogram for predicting 1-year, 3-year, and 5-year cancer-specific survival (CSS).

In a multivariate Cox regression model, after adjusting for age, laterality, number of tumours, tumour size, grading, T stage, N stage, examined N, chemotherapy, CEA, and PNI, a high mLNR remained positively correlated with poorer CSS (HR = 2.421, 95% CI = 2.126-2.757, P < 0.001; **Table 3**). Similar results were found in the validation cohort (HR = 2.531, 95% CI = 1.323-4.842, P = 0.005; **Supplementary Table 1**). The OS was consistent with the aforementioned results, with high mLNR continuing to demonstrate a positive association with poorer OS (training cohort: HR = 2.289, 95% CI = 2.040-2.569, P < 0.001; **Table 4**; validation cohort: HR = 2.618, 95% CI = 1.425-4.810, P = 0.002; **Supplementary Table 1**). Based on the multivariate Cox model, significant variables with p < 0.01 in the training cohort were incorporated into the final 1-, 3-, and 5-year CSS and OS prediction models, as illustrated in the nomograms (**Figure 4**; **Supplementary Figure 3**).

**Table 3:** Univariate and multivariate analysis of factors associated with cancer-specific survival in the training set.

Variables		Univariate analysis		Multivariate analysis	
		HR (95% CI)	P Value	HR (95% CI)	P Value
Sex					
	Male	1			
	Female	0.942 (0.897-0.990)	0.017		
Age (years)					
	< 65	1		1	
	≥ 65	1.949 (1.853-2.049)	< 0.001	1.580 (1.496-1.669)	< 0.001
Laterality					
	Left	1		1	
	Right	1.249 (1.231-1.360)	< 0.001	1.131 (1.073-1.193)	< 0.001
Tumor number					
	1	1		1	
	> 1	1.211 (1.148-1.279)	< 0.001	1.041 (0.985-1.100)	0.159
Size (cm)					
	< 5	1		1	
	≥ 5	1.345 (1.279-1.415)	< 0.001	1.107 (1.051-1.166)	< 0.001
Grade					
	Well/moderate	1		1	
	Poor/undifferentiated	1.717 (1.627-1.812)	< 0.001	1.339 (1.266-1.416)	< 0.001
T stage					
	T1-T2	1		1	
	T3-T4	2.847 (2.598-3.180)	< 0.001	2.060 (1.857-2.286)	< 0.001
N stage					
	N1	1		1	
	N2	1.818 (1.729-1.911)	< 0.001	1.496 (1.416-1.580)	< 0.001
Examined N					
	< 12	1		1	
	≥ 12	0.738 (0.692-0.788)	< 0.001	0.722 (0.675-0.772)	< 0.001
Chemotherapy					
	No	1		1	
	Yes	0.427 (0.406-0.449)	< 0.001	0.486 (0.461-0.513)	< 0.001
CEA					
	Negative	1		1	
	Positive	1.771 (1.686-1.861)	< 0.001	1.500 (1.427-1.577)	< 0.001
PNI					
	Absent	1		1	
	Present	1.717 (1.622-1.817)	< 0.001	1.435 (1.353-1.522)	< 0.001
mLNR					
	Low	1		1	
	Intermediate	1.911 (1.814-2.013)	< 0.001	1.519 (1.437-1.605)	< 0.001
	High	3.802 (3.363-4.298)	< 0.001	2.421 (2.126-2.757)	< 0.001

TD, tumor deposits; Examined N, total examined lymph nodes; CEA, carcino-embryonic antigen; PNI, perineural invasion; mLNR, modified lymph node ratio.

**Table 4:** Univariate and multivariate analysis of factors associated with overall survival in the training set.

Variables		Univariate analysis			Multivariate analysis	
		HR (95% CI)	P Value		HR (95% CI)	P Value
Sex						
	Male	1				
	Female	0.893 (0.858-0.929)		0.034		
Age (years)						
	< 65	1			1	
	≥ 65	2.606 (2.499-2.717)		< 0.001	1.963 (1.876-2.054)	< 0.001
Laterality						
	Left	1			1	
	Right	1.381 (1.327-1.437)		< 0.001	1.130 (1.084-1.179)	< 0.001
Tumor number						
	1	1			1	
	> 1	1.516 (1.454-1.580)		< 0.001	1.226 (1.175-1.280)	0.001
Size (cm)						
	< 5	1			1	
	≥ 5	1.262 (1.211-1.314)		< 0.001	1.074 (1.029-1.120)	< 0.001
Grade						
	Well/moderate	1			1	
	Poor/undifferentiated	1.565 (1.497-1.636)		< 0.001	1.243 (1.188-1.301)	< 0.001
T stage						
	T1-T2	1			1	
	T3-T4	1.973 (1.842-2.114)		< 0.001	1.546 (1.440-1.661)	< 0.001
N stage						
	N1	1			1	
	N2	1.456 (1.397-1.517)		< 0.001	1.322 (1.264-1.383)	< 0.001
Examined N						
	< 12	1			1	
	≥ 12	0.733 (0.696-0.772)		< 0.001	0.745 (0.706-0.786)	< 0.001
Chemotherapy						
	No	1			1	
	Yes	0.345 (0.331-0.359)		< 0.001	0.427 (0.409-0.445)	< 0.001
CEA						
	Negative	1			1	
	Positive	1.630 (1.566-1.695)		< 0.001	1.419 (1.363-1.477)	< 0.001
PNI						
	Absent	1			1	
	Present	1.429 (1.362-1.500)		< 0.001	1.301 (1.238-1.367)	< 0.001
mLNR						
	Low	1			1	
	Intermediate	1.587 (1.521-1.657)		< 0.001	1.370 (1.309-1.433)	< 0.001
	High	2.908 (2.606-3.245)		< 0.001	2.289 (2.040-2.569)	< 0.001

TD, tumor deposits; Examined N, total examined lymph nodes; CEA, carcino-embryonic antigen; PNI, perineural invasion; mLNR, modified lymph node ratio.

### Validation of the prediction models

The CSS prediction model yielded a C-index of 0.726 (95% CI, 0.714–0.737) in the training cohort and 0.779 (95% CI, 0.746–0.814) in the validation cohort. The corresponding C-indices for the OS model were 0.733 (95% CI, 0.724–0.742) and 0.767 (95% CI, 0.733–0.801), respectively.

**Figure 5:**
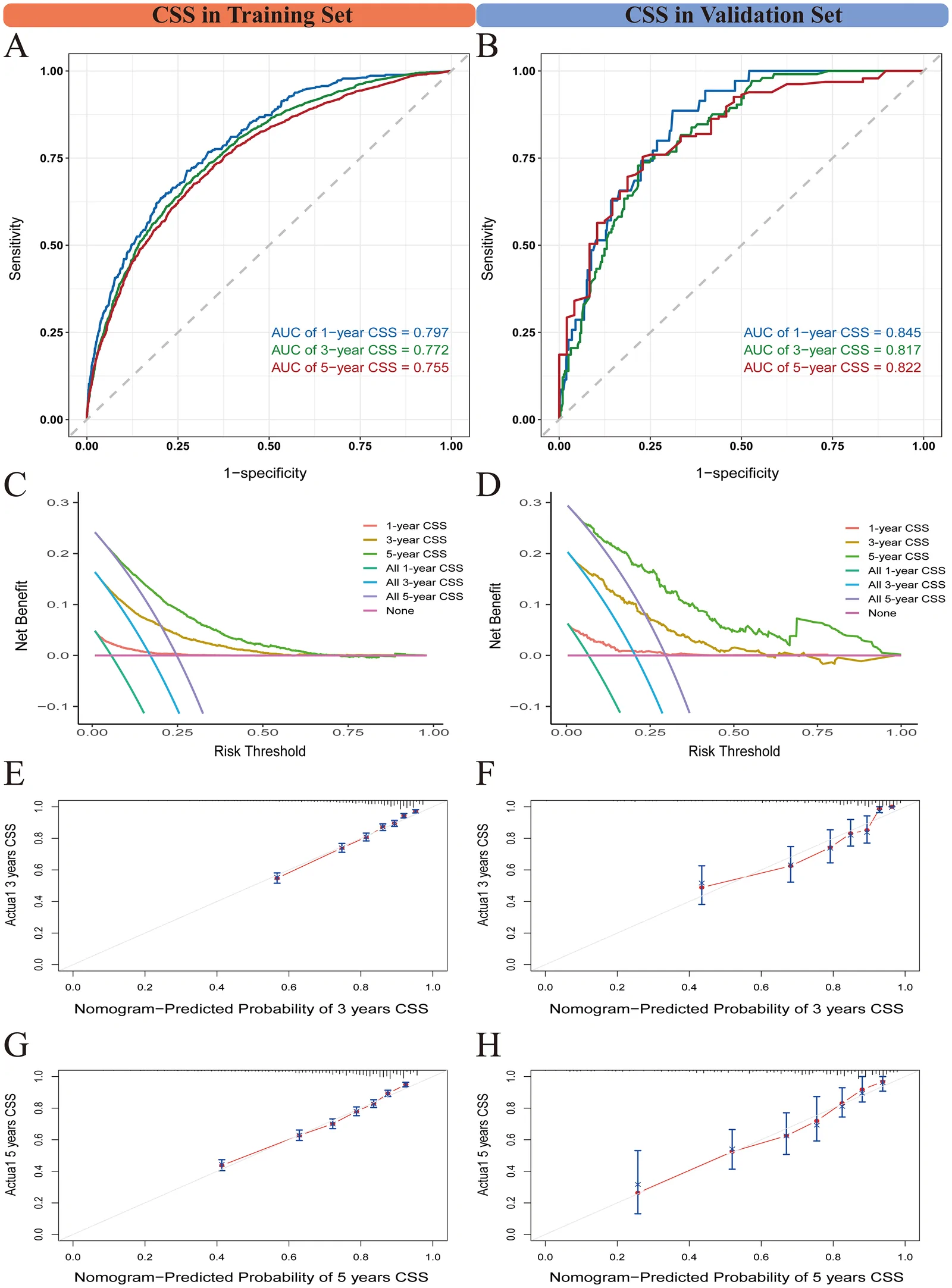
Predictive performance of the nomogram for cancer-specific survival (CSS) in training and validation sets. **(A, B)** ROC curves demonstrating the nomogram’s discrimination for 1-year, 3-year, and 5-year CSS in the training set **(A)** and validation set **(B)**, with corresponding AUC values; **(C, D)** Decision curve analysis (DCA) showing the net benefit of the nomogram for predicting 1-year, 3-year, and 5-year CSS at different risk thresholds in the training set **(C)** and validation set **(D)**; **(E, F)** Calibration plots assessing the accuracy of 3-year CSS predictions in the training set **(E)** and validation set **(F)**, comparing predicted probabilities with actual outcomes; **(G, H)** Calibration plots evaluating the consistency of 5-year CSS predictions in the training set **(G)** and validation set **(H)**, illustrating the agreement between predicted.

Time-dependent ROC analysis showed that both models had greater discriminatory ability than the individual prognostic factors. For CSS, the 1-, 3-, and 5-year AUCs were 0.797 (95% CI, 0.775–0.819), 0.772 (95% CI, 0.757–0.787), and 0.755 (95% CI, 0.741–0.770) in the training cohort (**Figure 5A**), and 0.845 (95% CI, 0.795–0.896), 0.817 (95% CI, 0.775–0.859), and 0.822 (95% CI, 0.748–0.895) in the validation cohort (**Figure 5B**). For OS, the corresponding AUCs were 0.806 (95% CI, 0.789–0.824), 0.775 (95% CI, 0.762–0.787), and 0.763 (95% CI, 0.751–0.776) in the training cohort (**Supplementary Figure 4A**), and 0.853 (95% CI, 0.803–0.886), 0.803 (95% CI, 0.761–0.845), and 0.817 (95% CI, 0.747–0.887) in the validation cohort (**Supplementary Figure 4B**).

DCA indicated that both models provided clinical net benefit (**Figures 5C, D**; **Supplementary Figures 4C, D**). Calibration plots also showed good agreement between the observed and predicted probabilities of CSS (**Figures 5E–H**) and OS (**Supplementary Figures 4E–H**).

## Discussion

This study showed that both TD status and TD count were associated with CSS and OS in patients with stage III CRC. Because the prognostic contribution of TD burden is not fully captured by conventional nodal staging, we integrated the TD-count and LNR categories to develop mLNR using a SEER-derived training cohort and evaluated it in an external validation cohort. mLNR improved prognostic stratification, and the mLNR-based nomogram showed consistent discrimination and calibration across the two cohorts. These findings support the consideration of TD burden alongside conventional nodal measures in postoperative risk assessment.

Gabriel et al. (22) first described TDs in 1935, and since then many studies have been carried out on the origin, distribution and clinical significance of TDs. For instance, Goldstein et al. (23) in 2000 emphasised their significance in adverse tumour outcomes and their influence on TNM classification. Based on these reports, the AJCC introduced TDs in 1997 in the 5th edition of the TNM staging system, which classified TDs based on size alone, with TDs >3 mm in diameter classified as regional LNM; TDs <3 mm in diameter is classified in the T category as a discontinuous extension, i.e., T3 (24). The definition of TDs has been revised in subsequent versions of the TNM staging system. In the last 2 versions of the TNM staging system (TNM7 and TNM8), TDs referred to discrete nodules of cancer in the lymph drainage area of pericolorectal adipose tissue, without histological evidence of neural structures, vessels, and lymph (25, 26). Staging is classified as N1c only in the presence of TDs (regardless of the number) in the absence of LNM. In contrast, if LNM is present, the presence of TDs is disregarded. The utilisation of the N1c parameter is the most significant advantage of the current TNM version, as it facilitates the classification of these tumours as stage III, necessitating adjuvant therapy (27). Nevertheless, the prognostic value of the number of TDs is severely underestimated in this case and the prognostic role of TDs is completely lost in the presence of LNM.

Despite the ambiguity surrounding the conceptualisation and categorisation of TDs within tumour staging systems, it is evident that TDs is associated with a poor prognosis in patients diagnosed with CRC. A population-based study suggests that the presence of both TDs and positive lymph nodes in stage III colon cancer is associated with poorer overall survival and that TDs has a similar prognostic impact as LNM (28). Another study showed that TDs was a poor prognostic factor in patients with stage III CRC, and that the co-existence of TDs and LNM was associated with the worst prognosis (29). It has been demonstrated in a number of other studies that TDs was a significant prognostic risk factor for cancer patients (30, 31). The aforementioned findings are consistent with our own, and moreover, we found that the presence of multiple TDs was also associated with reduced CSS and OS compared to a single TD. However, Liu et al. (32) found that the number of TDs was not associated with OS in their study. Nevertheless, it should be noted that a proportion of patients in the aforementioned study were classified as TNM stage IV or had liver metastases. This may have obscured the potential impact of TDs due to adverse factors associated with distant metastases, thereby rendering the number of TDs irrelevant to survival prognosis.

The present study posits that TDs possesses a high prognostic value, even when LNM is not factored into the equation. Furthermore, the correlation between the number of TDs and prognosis is significant. Tumour access to metastatic sites through multiple anatomical pathways leads to wider spread of the tumour, which increases the risk of metastatic spread and leads to poorer CSS (33). This partly explains why the prognosis is worse when TDs and LNM coexist than LNM alone. Therefore, the addition of TDs to LNM may help to identify patients at high risk for stage III CRC, which may further determine the modality and optimal duration of adjuvant therapy (34). In our study, mLNR, which integrates TD count and LNR, remained independently associated with CSS and OS after multivariable adjustment and effectively stratified patients into distinct prognostic groups. The mLNR-based nomogram showed consistent discrimination and calibration in the training and external validation cohorts, supporting its potential utility for postoperative risk assessment in patients with stage III CRC.

This study has several limitations. First, its retrospective design may have introduced selection bias and missing data, potentially limiting the representativeness and external validity of the findings. In addition, patients who received neoadjuvant therapy were excluded to ensure the comparability of postoperative pathological variables. Consequently, the rectal cancer subgroup represented a selected surgery-first population and may not reflect the full spectrum of stage III rectal cancer encountered in current clinical practice, particularly patients with locally advanced disease who routinely receive neoadjuvant therapy. Although the use of a SEER-derived training cohort and an external Chinese hospital cohort partly supported the generalisability of the findings, this potential selection bias remains. Second, TDs were classified as absent, single, or multiple rather than analysed according to their exact count. Future studies should evaluate the full TD count and its interactions with other clinical features. Finally, the mechanisms underlying the prognostic value of mLNR remain unclear. Prospective studies are needed to determine whether dynamic changes in TDs and LNR can inform treatment decisions and facilitate individualised management of CRC.

## Conclusions

TD status, TD count, and LNR were independently associated with CSS and OS in patients with stage III CRC. Their integration into mLNR improved prognostic stratification, and the mLNR-based nomogram showed consistent discrimination and calibration for predicting CSS and OS in both the training and validation cohorts. This model may support postoperative risk assessment and clinical decision-making in patients with stage III CRC.

## Data Availability

The publicly available SEER data used in this study can be accessed from the Surveillance, Epidemiology, and End Results (SEER) Program of the National Cancer Institute at https://seer.cancer.gov/data/. Repository: SEER Program, National Cancer Institute. Accession number: not applicable. Access to SEER data requires completion of the SEER data access request/data-use agreement and use of SEER*Stat.

## Supplementary material

Supplementary Figure 1 Kaplan-Meier curves for overall survival (OS) stratified by different biomarkers in the training set **(A–D)** and validation set **(E-H)**. **(A, E)** Kaplan-Meier curves stratified by modified lymph node ratio (mLNR); **(B, F)** Kaplan-Meier curves stratified by lymph node ratio (LNR); **(C, G)** Kaplan-Meier curves stratified by the presence or absence of tumor deposits (TD; binary classification, TD2); **(D, H)** Kaplan-Meier curves stratified by the number of tumor deposits (TD: 0, 1, or >1; ternary classification, TD3). TD represents tumor deposits.

Supplementary Figure 2 Kaplan-Meier curves stratified by single TD and multiple TDs in the training set **(A, B)** and validation set **(C, D)**. **(A, C)** Kaplan-Meier curves for cancer-specific survival (CSS); **(B, D)** Kaplan-Meier curves for overall survival (OS).

Supplementary Figure 3 Nomogram for predicting 1-year, 3-year, and 5-year overall survival (OS).

Supplementary Figure 4 Predictive performance of the nomogram for overall survival (OS) in training and validation sets. (A, B) ROC curves demonstrating the nomogram’s discrimination for 1-year, 3-year, and 5-year OS in the training set **(A)** and validation set **(B)**, with corresponding AUC values; **(C, D)** Decision curve analysis (DCA) showing the net benefit of the nomogram for predicting 1-year, 3-year, and 5-year OS at different risk thresholds in the training set **(C)** and validation set **(D)**; **(E, F)** Calibration plots assessing the accuracy of 3-year OS predictions in the training set **(E)** and validation set **(F)**, comparing predicted probabilities with actual outcomes; (G, H) Calibration plots evaluating the consistency of 5-year OS predictions in the training set **(G)** and validation set **(H)**, illustrating the agreement between predicted.
