# Supplementary material for: Prognostic value of a modified lymph node ratio for combined colorectal cancer tumour deposits: a population-based study
Source: Front Oncol. 2026 Sep 3;16:1910628. doi: 10.3389/fonc.2026.1910628 (PMC13581868; doi:10.3389/fonc.2026.1910628)
Supplement: Supplementary file 1 [file DataSheet1.docx]

**Figure S1** **Kaplan-Meier curves for** **overall survival (OS) stratified by different biomarkers in the training set (A-D) and validation set (E-H).**

(A, E) Kaplan-Meier curves stratified by modified lymph node ratio (mLNR); (B, F) Kaplan-Meier curves stratified by lymph node ratio (LNR); (C, G) Kaplan-Meier curves stratified by the presence or absence of tumor deposits (TD; binary classification, TD2); (D, H) Kaplan-Meier curves stratified by the number of tumor deposits (TD: 0, 1, or >1; ternary classification, TD3). TD represents tumor deposits.

**Figure S2 Kaplan-Meier curves stratified by single TD and multiple TDs in the training set (A, B) and validation set (C, D).**

(A, C) Kaplan-Meier curves for cancer-specific survival (CSS); (B, D) Kaplan-Meier curves for overall survival (OS).

**Figure S3 Nomogram for predicting 1-year, 3-year, and 5-year overall survival (OS).**

**Figure S4 Predictive performance of the nomogram for overall survival (OS) in training and validation sets.**

(A, B) ROC curves demonstrating the nomogram’s discrimination for 1-year, 3-year, and 5-year OS in the training set (A) and validation set (B), with corresponding AUC values; (C, D) Decision curve analysis (DCA) showing the net benefit of the nomogram for predicting 1-year, 3-year, and 5-year OS at different risk thresholds in the training set (C) and validation set (D); (E, F) Calibration plots assessing the accuracy of 3-year OS predictions in the training set (E) and validation set (F), comparing predicted probabilities with actual outcomes; (G, H) Calibration plots evaluating the consistency of 5-year OS predictions in the training set (G) and validation set (H), illustrating the agreement between predicted.
