# Supplementary material for: Prognostic value of a modified lymph node ratio for combined colorectal cancer tumour deposits: a population-based study
Source: Front Oncol. 2026 Sep 3;16:1910628. doi: 10.3389/fonc.2026.1910628 (PMC13581868; doi:10.3389/fonc.2026.1910628)
Supplement: Supplementary file 2 [file Table1.docx]

| **Supplemental Table1** Multivariable analyses of overall survival and cancer-specific survival in the validation set. | | | | | | |
| --- | --- | --- | --- | --- | --- | --- |
| **Variables** | | **CSS** | |  | **OS** | |
|  |  | **HR (95%CI)** | **P Value** |  | **HR (95%CI)** | **P Value** |
| Age (years) |  |  |  |  |  |  |
|  | < 65 | 1 |  |  | 1 |  |
|  | ≥ 65 | 1.798 (1.236-2.615) | 0.002 |  | 1.843 (1.303-2.607) | 0.001 |
| Laterality |  |  |  |  |  |  |
|  | Left | 1 |  |  | 1 |  |
|  | Right | 1.167 (0.775-1.756) | 0.460 |  | 1.302 (0.900-1.885) | 0.162 |
| Size (cm) |  |  |  |  |  |  |
|  | < 5 | 1 |  |  | 1 |  |
|  | ≥ 5 | 1.335 (0.874-2.038) | 0.181 |  | 1.343 (0.908-1.986) | 0.140 |
| Grade |  |  |  |  |  |  |
|  | Well/moderate | 1 |  |  | 1 |  |
|  | Poor/undifferentiated | 1.325 (0.788-2.228) | 0.289 |  | 1.249 (0.766-2.039) | 0.373 |
| T stage |  |  |  |  |  |  |
|  | T1-T2 | 1 |  |  | 1 |  |
|  | T3-T4 | 2.471 (1.065-5.732) | 0.035 |  | 2.939 (1.273-6.784) | 0.012 |
| N stage |  |  |  |  |  |  |
|  | N1 | 1 |  |  | 1 |  |
|  | N2 | 1.469 (0.984-2.194) | 0.060 |  | 1.295 (0.889-1.886) | 0.178 |
| Examined N |  |  |  |  |  |  |
|  | < 12 | 1 |  |  | 1 |  |
|  | ≥ 12 | 0.584 (0.367-0.929) | 0.023 |  | 0.578 (0.375-0.892) | 0.013 |
| Chemotherapy |  |  |  |  |  |  |
|  | No | 1 |  |  | 1 |  |
|  | Yes | 0.430 (0.294-0.630) | < 0.001 |  | 0.449 (0.316-0.639) | < 0.001 |
| CEA |  |  |  |  |  |  |
|  | Negative | 1 |  |  | 1 |  |
|  | Positive | 2.593 (1.755-3.829) | < 0.001 |  | 2.325 (1.630-3.316) | < 0.001 |
| PNI |  |  |  |  |  |  |
|  | Absent | 1 |  |  | 1 |  |
|  | Present | 1.465 (1.020-2.106) | 0.039 |  | 1.503 (1.075-2.100) | 0.017 |
| mLNR |  |  |  |  |  |  |
|  | Low | 1 |  |  | 1 |  |
|  | Intermediate | 2.001 (1.345-2.977) | 0.001 |  | 1.865 (1.290-2.696) | 0.001 |
|  | High | 2.531 (1.323-4.842) | 0.005 |  | 2.618 (1.425-4.810) | 0.002 |
| Abbreviations: CSS, cancer-specific survival; OS, overall survival; TD, tumor deposits; Examined N, total examined lymph nodes; CEA, carcino-embryonic antigen; PNI, perineural invasion; mLNR, modified lymph node ratio. | | | | | | |
